# Electroacupuncture in the treatment of non-alcoholic fatty liver disease: mechanistic insights and therapeutic potential

**DOI:** 10.3389/fmed.2026.1783519

**Published:** 2026-04-24

**Authors:** Yi Wang, Lai Zhang

**Affiliations:** 1 Department of Rehabilitation, Taizhou Central Hospital (Taizhou University Hospital), Taizhou, Zhejiang, China; 2 Department of Orthopedics, Taizhou Municipal Hospital, Taizhou, Zhejiang, China

**Keywords:** electroacupuncture, insulin resistance, lipid metabolism, non-alcoholic fatty liver disease, systematic review

## Abstract

Non-alcoholic fatty liver disease (NAFLD) has become the most prevalent chronic liver disorder globally, driven by increasing rates of obesity, insulin resistance, and metabolic dysfunction. In the absence of approved pharmacological therapies, electroacupuncture (EA) has garnered attention as a promising adjunctive intervention. This review synthesizes recent advances in understanding the mechanistic underpinnings and therapeutic potential of EA in NAFLD. We discuss how EA modulates hepatic lipid metabolism, insulin signaling, inflammation, fibrosis, and autophagy through key molecular pathways, including AMPK, SIRT1, PI3K/Akt, and NF-κB. Preclinical studies and emerging clinical trials indicate that EA effectively improves hepatic steatosis, metabolic parameters, and liver function with minimal side effects. Collectively, these findings underscore EA as a promising strategy for NAFLD treatment and highlight the need for further translational research.

## Introduction

Metabolic dysfunction-associated steatotic liver disease (MASLD), formerly known as non-alcoholic fatty liver disease (NAFLD), has become the most prevalent chronic liver disorder globally (1–3). Following a multi-society Delphi consensus in 2023, the nomenclature was updated to more accurately reflect the underlying metabolic pathophysiology and reduce stigma associated with prior terminology (4, 5). The disease spectrum now encompasses metabolic dysfunction-associated steatotic liver (MASL) and metabolic dysfunction-associated steatohepatitis (MASH), the latter characterized by hepatic inflammation and hepatocellular injury with or without fibrosis (2, 4). While the term MAFLD (metabolic dysfunction-associated fatty liver disease) was previously proposed, the international consensus has adopted MASLD as the standard nomenclature (5, 6). MASLD encompasses a spectrum of hepatic abnormalities, ranging from simple steatosis and non-alcoholic steatohepatitis (NASH) to cirrhosis and hepatocellular carcinoma (7). Its global prevalence is steadily increasing, currently affecting approximately one-third of the adult population (8). Notably, patients often remain asymptomatic until advanced stages such as cirrhosis, contributing to the disease’s silent progression and rising incidence (9). Complications associated with MASLD further exacerbate disease severity. Therefore, extensive clinical and preclinical studies are urgently needed to elucidate its pathogenesis and provide a robust theoretical basis for the development of novel therapeutic strategies.

The pathophysiology of MASLD is multifactorial and closely linked to obesity, metabolic syndrome, insulin resistance, and type 2 diabetes mellitus (T2DM) (10). Its development follows a “multiple-hit” model characterized by hepatic steatosis, oxidative stress, mitochondrial dysfunction, inflammation, and fibrosis (11, 12). Central molecular mechanisms include dysregulated lipid metabolism, cytokine imbalances, and impaired autophagy. Abnormal activation of transcription factors such as sterol regulatory element-binding protein 1c (SREBP-1c) and peroxisome proliferator-activated receptor gamma (PPARγ) promotes lipogenesis, while reduced fatty acid oxidation aggravates hepatic lipid accumulation (13). Chronic low-grade inflammation, mediated by tumor necrosis factor-alpha (TNF-α), interleukin-6 (IL-6), and NLRP3 inflammasomes, contributes to hepatocellular injury and fibrogenesis (14, 15). In individuals with obesity and metabolic syndrome, excessive caloric intake and sedentary behavior drive adipose tissue dysfunction and elevate circulating free fatty acids (FFAs), which are taken up by the liver and converted into triglycerides via enhanced lipogenesis (16). Insulin resistance-a hallmark of metabolic syndrome and T2DM-impairs insulin signaling, leading to insufficient suppression of gluconeogenesis and upregulation of SREBP-1c, thereby exacerbating steatosis (17). Concurrent hyperinsulinemia and chronic inflammation, mediated by adipokines and pro-inflammatory cytokines such as TNF-α and IL-6, further impair hepatic insulin sensitivity and promote oxidative stress and mitochondrial damage (18). These insults induce lipid peroxidation, endoplasmic reticulum stress, and hepatocyte apoptosis, ultimately driving MASLD progression.

Currently, managing MASLD remains a major clinical challenge, as no pharmacological agents have been specifically approved for its treatment. Lifestyle interventions-including dietary modifications, weight loss, and increased physical activity-remain the cornerstone of therapy. Sustained weight loss of 7%–10% has been shown to improve steatosis, inflammation, and even fibrosis (19). However, long-term adherence to lifestyle modifications is often limited. Various pharmacological strategies are under investigation. Insulin sensitizers such as pioglitazone and GLP-1 receptor agonists (e.g., liraglutide and semaglutide) have demonstrated efficacy in improving steatosis in patients with MASH (20). In a major breakthrough, resmetirom–a thyroid hormone receptor-β (THR-β) agonist–became the first pharmacologic agent approved by the U.S. Food and Drug Administration (FDA) for the treatment of non-cirrhotic MASH with moderate to advanced liver fibrosis, marking a pivotal milestone in the field (4, 21). Beyond resmetirom, the therapeutic landscape continues to expand rapidly. Novel agents targeting diverse molecular pathways are advancing through clinical development, including fibroblast growth factor 21 (FGF21) analogs, pan-PPAR agonists, and FXR agonists (22). Notably, DR10624–a first-in-class long-acting triagonist targeting FGF21R, GCGR, and GLP-1R–has recently completed patient enrollment in Phase II trials for MASLD/MASH, with top-line data anticipated in late 2026. Emerging therapeutic targets identified through bioinformatics and Mendelian randomization analyses, such as UHRF1, are also under preclinical investigation (23). Additionally, innovative approaches including FABP1 inhibitors and nanoparticle-based agents (e.g., the carboxyl fullerene derivative QF70) have shown promise in preclinical MASLD models by reducing hepatic lipid accumulation, inflammation, and fibrosis (23, 24). Despite these developments, therapeutic outcomes remain inconsistent for many agents, and long-term safety data beyond resmetirom are still lacking. Recent preclinical and clinical studies suggest that electroacupuncture (EA) may exert beneficial effects on hepatic steatosis, inflammation, fibrosis, and metabolic dysfunction via multiple molecular mechanisms.

Recent preclinical and clinical studies suggest that electroacupuncture (EA) may exert beneficial effects on hepatic steatosis, inflammation, fibrosis, and metabolic dysfunction via multiple molecular mechanisms.

Electroacupuncture, a modern refinement of traditional acupuncture, delivers low-frequency electrical stimulation through needles inserted into specific acupoints, thereby enhancing the consistency and reproducibility of stimulation (25). Biologically, EA has been shown to modulate neural, hormonal, and immune responses (26, 27). It influences autonomic nervous system activity, promotes endogenous opioid release, alters cytokine profiles, and regulates key metabolic signaling pathways, including AMP-activated protein kinase (AMPK), phosphoinositide 3-kinase/Akt (PI3K/Akt), and sirtuin 1 (SIRT1) (28, 29). These effects position EA as a promising modality for addressing systemic metabolic dysfunction, including that observed in MASLD. Nevertheless, the precise mechanisms through which EA confers its therapeutic effects in MASLD remain incompletely understood, necessitating further in-depth investigations. This review aims to synthesize current mechanistic insights into EA and explore its therapeutic potential in the context of MASLD.

## Mechanisms of EA in the regulation of hepatic lipid metabolism

Electroacupuncture has emerged as a promising therapeutic strategy for metabolic disorders, particularly those involving hepatic lipid dysregulation. A growing body of evidence indicates that EA exerts multifaceted regulatory effects on liver lipid metabolism by modulating lipid synthesis, oxidation, and systemic energy homeostasis through complex molecular and neuroendocrine mechanisms.

A primary mechanism by which EA regulates hepatic lipid metabolism involves the activation of the AMP-activated protein kinase (AMPK) signaling pathway (30). As a sensor of cellular energy, AMPK activation promotes fatty acid oxidation while inhibiting lipogenesis (31). In various models of diet-induced obesity (DIO), polycystic ovary syndrome (PCOS), and genetically obese db/db mice, EA at acupoints such as ST36, CV3, and ST40 has been shown to restore phosphorylated AMPK (p-AMPK) levels suppressed by metabolic dysregulation (32–34). This AMPK activation is accompanied by decreased phosphorylation of its downstream target, acetyl-CoA carboxylase (ACC), thereby inhibiting malonyl-CoA production and enhancing mitochondrial fatty acid oxidation (33). Moreover, EA upregulates the expression of carnitine palmitoyltransferase-1 (CPT-1), a rate-limiting enzyme in mitochondrial fatty acid import, further promoting lipid catabolism (33).

In addition to the AMPK–ACC axis, EA also influences lipid biosynthesis via modulation of the mechanistic target of rapamycin (mTOR) signaling pathway. In hyperlipidemic rats, EA at the Fenglong (ST40) acupoint suppressed the expression of phosphorylated mTOR (p-mTOR) and its associated deubiquitinase USP20 (35). The mTOR–USP20 axis stabilizes 3-hydroxy-3-methylglutaryl coenzyme A reductase (HMGCR), the rate-limiting enzyme in cholesterol biosynthesis, by preventing its ubiquitin-mediated degradation (35, 36). EA attenuates this stabilization, leading to decreased HMGCR protein levels and suppression of hepatic cholesterol synthesis (35). These findings suggest that EA exerts post-translational control over key enzymes involved in lipid metabolism.

Electroacupuncture has also demonstrated efficacy in improving systemic insulin sensitivity and hepatic insulin signaling, which are closely linked to lipid metabolic homeostasis (37). In a PCOS model characterized by insulin resistance and hepatic steatosis, EA significantly improved glucose tolerance and reduced fasting insulin levels (38). Mechanistically, these effects were associated with restoration of the phosphatidylinositol 3-kinase (PI3K)/Akt signaling pathway, as indicated by increased levels of phosphorylated Akt (Ser473), glucose transporter 4 (GLUT4), and phosphorylated glycogen synthase kinase 3β (GSK3β) (38). Restoration of these signaling components facilitated glucose uptake and suppressed adipogenic gene expression, implying that EA indirectly mitigates hepatic lipid accumulation by enhancing insulin responsiveness (38). Furthermore, EA has been shown to augment adiponectin signaling-a pathway known for its anti-lipogenic and insulin-sensitizing properties (34). In obese PCOS rats, EA increased expression of adiponectin and its downstream effectors, liver kinase B1 (LKB1) and AMPKα1, while concurrently reducing ACC expression, suggesting that EA restores systemic metabolic balance through adipokine-mediated signaling (34).

A particularly compelling aspect of EA’s mechanism involves neuromodulation and the regulation of the liver–brain axis (39, 40). In a model of olanzapine-induced metabolic dysfunction, EA at ST36 ameliorated dyslipidemia and hepatic steatosis while normalizing the disrupted circadian respiratory exchange ratio (41). Using functional ultrasound imaging, retrograde viral tracing, and c-Fos mapping, researchers observed significant inhibition of agouti-related peptide (AGRP)-expressing neurons in the lateral septal nucleus (LS), nucleus accumbens (NAc), and white matter tracts, indicating that EA modulates specific central circuits involved in energy homeostasis and hepatic function (41). These findings support that EA affects hepatic lipid metabolism through central nervous system pathways.

In addition to the typical metabolic pathways, emerging evidence suggests that EA may modulate novel molecular signaling axes in the liver, although these findings remain primarily at the preclinical stage. Transcriptomic profiling of ovariectomized, high-fat diet-fed (OVX + HFD) rats identified the Pdia3/Perk/Qrich1 signaling cascade as a potential mediator of EA’s metabolic benefits (42). EA reversed the upregulation of protein disulfide-isomerase A3 (Pdia3) and its downstream targets, Perk and Qrich1-factors associated with endoplasmic reticulum (ER) stress and lipid biosynthesis (42). These results suggest that EA alleviates hepatic lipid accumulation by mitigating ER stress–related lipogenic signaling. However, it is important to emphasize that this pathway has only been investigated in animal models, and its clinical relevance in human NAFLD/MASLD remains to be established. Future studies employing human liver tissues or translational approaches are warranted to validate whether Pdia3/Perk/Qrich1 signaling contributes to EA’s therapeutic effects in patients.

From a histopathological standpoint, EA consistently reduces hepatic steatosis across multiple animal models (32–34). This is evidenced by reductions in liver weight, hepatic triglyceride and cholesterol content, and histological indicators of lipid droplet accumulation (32–34). Concurrent improvements in systemic lipid profiles–including decreased serum levels of total cholesterol, low-density lipoprotein cholesterol (LDL-C), triglycerides, and free fatty acids-further highlight EA’s therapeutic potential in mitigating hepatic lipid overload (32–34).

Importantly, these diverse signaling pathways do not function in isolation but rather constitute an integrated regulatory network through which EA exerts its metabolic benefits. For instance, the AMPK/SIRT1 axis serves as a central hub connecting energy sensing with anti-inflammatory and pro-autophagic responses (43). AMPK activation enhances SIRT1 activity via NAD+ modulation, leading to coordinated regulation of downstream targets including PGC-1α, FOXO, and NF-κB. Recent evidence demonstrates that EA at CV4, BL23, ST40, and ST25 activates the AMPK/SIRT1 pathway, promoting white adipose tissue browning and improving systemic lipid metabolism through upregulation of neuregulin 4 (Nrg4) (43). This crosstalk between AMPK and SIRT1 exemplifies how EA orchestrates multiple pathways to achieve synergistic therapeutic effects. Furthermore, the PI3K/Akt pathway interacts with AMPK signaling through TSC2-mediated regulation of mTORC1, creating a feedback loop that fine-tunes cellular energy homeostasis. The NF-κB pathway, while primarily mediating inflammatory responses, is modulated by both SIRT1-dependent deacetylation and AMPK-mediated metabolic control, illustrating the convergence of metabolic and immune signaling. Thus, EA operates through a “neuro-endocrine-immune-metabolic” network, wherein peripheral stimulation at specific acupoints triggers integrated central and peripheral responses that collectively ameliorate hepatic steatosis and systemic metabolic dysfunction (44).

**FIGURE 1:**
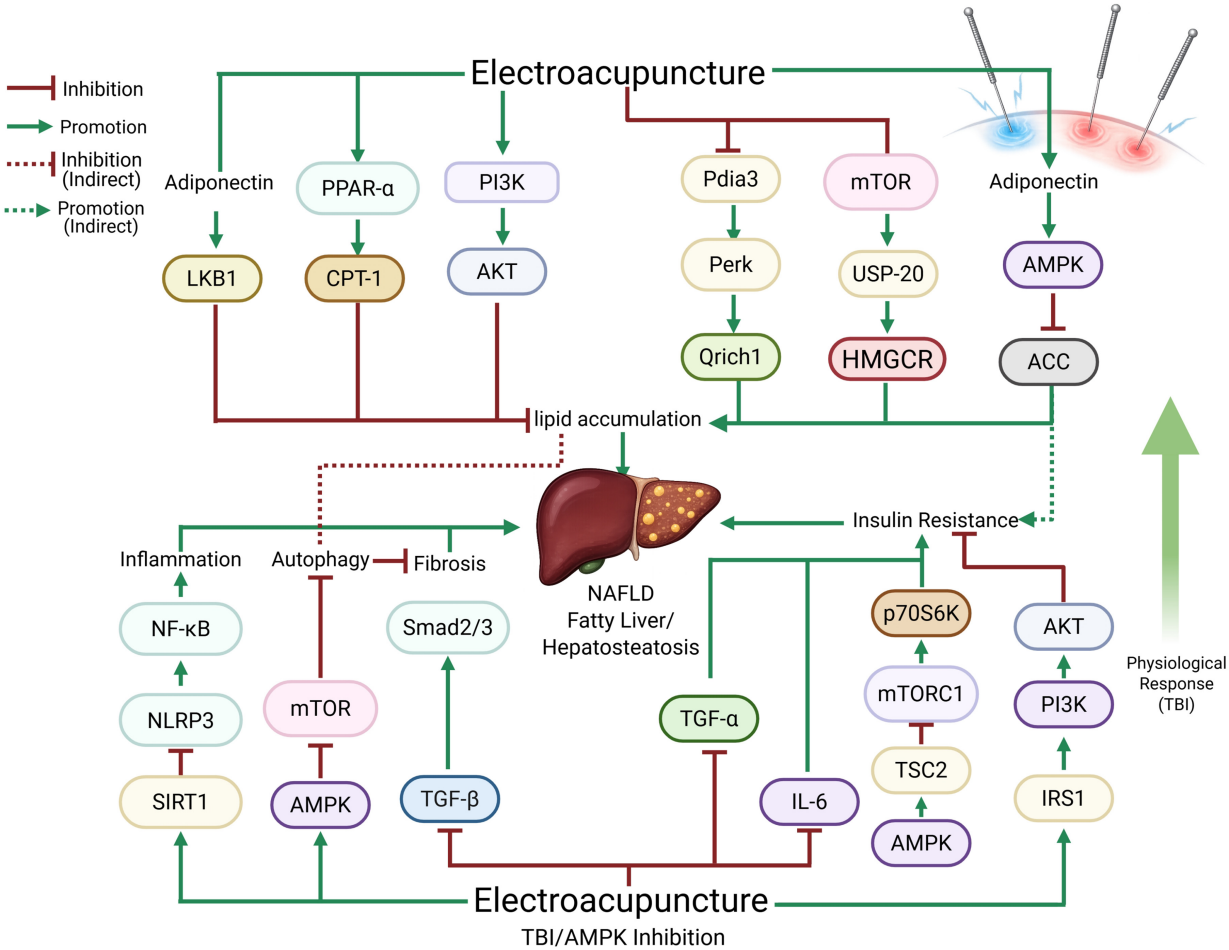
Pathway diagram of the molecular mechanism by which electroacupuncture improves NAFLD. The system showed the complete regulatory network of EA intervention on NAFLD through multi-targets and multi-signaling pathways.

Collectively, these findings depict a complex and integrative picture of EA-mediated regulation of hepatic lipid metabolism. EA acts through multiple pathways-AMPK/ACC activation, mTOR/HMGCR suppression, PI3K/Akt restoration, ER stress mitigation, and brain-liver axis modulation-to achieve a net reduction in hepatic lipid synthesis and storage. Figure 1 shows the pathway diagram of the molecular mechanism by which electroacupuncture improves NAFLD. These mechanistic insights, primarily derived from robust preclinical models, offer a convincing theoretical basis for future translational studies aimed at validating EA as a non-pharmacologic intervention for MASLD, obesity-related liver dysfunction, and metabolic syndrome.

## Modulation of insulin resistance and glucose homeostasis by EA

Insulin resistance is a hallmark of MASLD and a critical driver of its progression (45). EA enhances both systemic and hepatic insulin sensitivity through multiple mechanisms. Preclinical studies using genetic and diet-induced models of MASLD consistently demonstrate that EA ameliorates insulin resistance and restores glucose homeostasis (32, 46). In Zucker diabetic fatty (ZDF) rats, a model characterized by spontaneous obesity, hepatic steatosis, and insulin resistance, EA significantly reduced fasting plasma glucose, insulin, C-peptide levels, and the homeostatic model assessment for insulin resistance (HOMA-IR) index (47). These metabolic improvements were accompanied by histological restoration of hepatic architecture, including reduced hepatocyte ballooning and inflammatory infiltration (48–50). Mechanistically, EA reestablishes hepatic energy balance by modulating the AMP-activated protein kinase (AMPK)/mechanistic target of rapamycin complex 1 (mTORC1)/ribosomal protein S6 kinase (p70S6K) signaling axis (47). Upregulation of phosphorylated AMPK and its downstream target tuberous sclerosis complex 2 (TSC2) was observed in hepatocytes, indicating enhanced energy sensing and suppressed anabolic signaling (47). Meanwhile, downregulation of mTORC1 and p70S6K, both of which are associated with insulin desensitization, highlighted the capacity of EA to restore insulin responsiveness (47).

The insulin-sensitizing effects of EA are also attributed to the activation of canonical insulin signaling pathways, particularly the IRS1/PI3K/AKT cascade. In high-fat diet (HFD) and letrozole-induced polycystic ovary syndrome (PCOS) rat models-conditions frequently coexisting with MASLD-EA enhanced the expression of insulin receptor substrate-1 (IRS1), phosphatidylinositol 3-kinase (PI3K), and protein kinase B (AKT) (38). This led to improved insulin-stimulated glucose uptake and increased phosphorylation of downstream effectors such as glycogen synthase kinase 3 beta (GSK3β) and glucose transporter 4 (GLUT4), particularly in hepatic and skeletal muscle tissues (38). These molecular changes were consistent with the observed reduction in fasting glucose, insulin levels, and HOMA-IR values, suggesting that EA not only improves systemic insulin sensitivity but also promotes glycogen storage, thus reestablishing glucose equilibrium (38).

Anti-inflammatory mechanism has also been implicated directly in the insulin-sensitizing actions of EA (51). In MASLD models, EA downregulated hepatic mRNA expression of proinflammatory cytokines, including tumor necrosis factor-alpha (TNF-α) and interleukin-6 (IL-6), which disrupt insulin receptor signaling via serine phosphorylation of IRS1 (38, 52). Meanwhile, EA promotes the expression of PPAR-α, a nuclear receptor involved in fatty acid oxidation and anti-inflammatory regulation in the liver (38, 53, 54). These results indicate that EA alleviates MASLD through overlapping anti-inflammatory and insulin-sensitizing effects.

A pivotal mechanism underlying EA’s systemic metabolic effects involves its regulation of adipokine secretion and adipose tissue function. Adiponectin, an insulin-sensitizing adipokine inversely correlated with MASLD severity, is markedly upregulated following EA treatment (34, 55). The elevated adiponectin level is associated with increased hepatic expression of AMPK and liver kinase B1 (LKB1), suggesting that the adiponectin-AMPK axis is a central mediator of EA’s glucose-lowering effect (56, 57). In contrast, EA reduced hepatic ACC expression and activity, thereby attenuating gluconeogenesis and adipogenesis, both of which are exaggerated under insulin-resistant conditions (34). These findings suggest that EA reprograms hepatic glucose metabolism by promoting insulin-dependent glucose utilization while suppressing excessive glucose production.

Emerging evidence also implicates the critical role of gut-liver axis modulation in EA-induced glycemic improvements (58). In Zucker diabetic rats, EA significantly altered the composition of gut microbiota, increasing microbial diversity and abundance of beneficial taxa such as Adlercreutzia, which has been correlated with improved insulin sensitivity (46). Metabolomic analyses further revealed correlations between microbial metabolites (e.g., allantoin) and hepatic glucose metabolic profiles, indicating that EA may exert hypoglycemic effect partly through microbiota-derived signaling pathways (46). More recent studies have elucidated specific molecular mechanisms underlying EA’s modulation of the gut-liver axis. Wang et al. demonstrated that EA at ST-36 attenuates intestinal barrier disruption in a mouse model of MAFLD via the α7nAChR-mediated HO-1/p38 MAPK/NF-κB pathway (44). EA treatment upregulated tight junction proteins (ZO-1, Occludin, Claudin-1), reduced gut permeability, and decreased circulating endotoxin levels, thereby alleviating hepatic inflammation and fibrosis. These protective effects were abolished by α7nAChR inhibition or intestinal-specific HO-1 knockout, confirming the mechanistic dependence on this cholinergic anti-inflammatory pathway (44). This gut-centric mechanism positions EA as a multimodal intervention that not only directly targets hepatic metabolism but also preserves intestinal barrier integrity, reducing the translocation of pro-inflammatory microbial products that drive MASLD progression. The above-mentioned research indicates that EA inhibits the progression of MASLD by improving liver insulin sensitivity through the gut-liver axis.

Furthermore, EA has been shown to remodel hepatic mitochondrial function, another crucial determinant of metabolic homeostasis in MASLD (59, 60). Transmission electron microscopy of hepatocytes from EA-treated ZDF rats revealed restoration of mitochondrial morphology, suggesting improved mitochondrial respiration and reduced oxidative stress (47). This is particularly relevant as mitochondrial dysfunction contributes to hepatic insulin resistance by impairing ATP production and increasing reactive oxygen species, thereby inhibiting insulin signaling (61, 62).

The involvement of AMPK/ACC signaling in EA’s regulation of glucose metabolism has been further validated in genetically obese db/db mice (32, 63). Inhibition of AMPK or ACC abrogated EA-mediated improvements in serum glucose, insulin levels, and hepatic glycogen synthesis, suggesting that the metabolic benefits of EA are, at least in part, partially dependent on this signaling pathway (32). These findings emphasize the central position of the AMPK pathway in mediating EA’s systemic actions on glucose homeostasis and support its use as a therapeutic target for MASLD.

Taken together, these preclinical findings paint a compelling picture that EA is a multimodal regulator of insulin sensitivity and glucose metabolism in MASLD. Through coordinated actions on energy-sensing pathways (AMPK/mTOR), insulin signaling (IRS1/PI3K/AKT), adipokine regulation, mitochondrial function, inflammatory responses, and the gut microbiome, EA significantly improves insulin resistance and inhibits the progression of MASLD.

## EA effects on inflammation, fibrosis, and autophagy in MASLD

Beyond its classical involvement in hepatic lipid accumulation and insulin resistance, the progression of MASLD is closely associated with chronic hepatic inflammation, fibrogenesis, and dysregulated autophagy (64–66). Accumulating experimental evidence indicates that EA exerts therapeutic effects on MASLD by targeting molecular pathways governing inflammatory signaling, fibrotic remodeling, and autophagic flux.

In rodent models of MASLD induced by a high-fat diet (HFD), EA significantly reduced the levels of serum alanine aminotransferase (ALT) and aspartate aminotransferase (AST), demonstrating that it significantly alleviated liver injury (67). Histopathological analysis further confirmed these findings, showing a reduction in steatosis, hepatocyte ballooning and inflammatory infiltration in animals treated with EA (67). Mechanistically, EA downregulated hepatic proinflammatory cytokines and inhibited activation of the nuclear factor kappa B (NF-κB) pathway, a central regulator of innate immunity and inflammation in NASH pathogenesis (44, 68). Concomitantly, EA upregulated the expression of SIRT1, a NAD+ -dependent histone deacetylase implicated in metabolic homeostasis and anti-inflammatory responses (69, 70). Notably, SIRT1 negatively regulates the NLRP3 inflammasome and NF-κB transcriptional activity, suggesting that the liver-protective effect of EA may be mediated via modulation of the SIRT1/NF-κB axis (67). These findings underscore EA’s capacity to suppress hepatic inflammation through epigenetic and signaling-based mechanisms, ultimately inhibiting MASLD progression.

In addition to its anti-inflammatory properties, EA also exhibits anti-fibrotic effects (71, 72). In T2DM-associated MASLD models, EA significantly improved liver histomorphology, decreased collagen deposition, and reduced fibrotic scores (73). These effects are attributed to the inhibition of the EA-mediated transforming growth factor-β1 (TGF-β1)/Smad2/3 signaling pathway, which is a typical driver of hepatic stellate cell activation and extracellular matrix (ECM) synthesis (73, 74). The attenuation of fibrogenesis was further supported by decreased expression of α-smooth muscle actin (α-SMA) and collagen type I in liver tissue following EA treatment (73). Notably, these anti-fibrotic effects were amplified when EA was combined with metformin, highlighting the potential of EA as a synergistic component in integrative therapeutic strategies for MASLD (73).

Autophagy, a cellular process responsible for the degradation and recycling of intracellular components, plays a critical role in lipid droplet clearance, mitochondrial quality control, and modulation of inflammatory responses in hepatocytes (75–77). In MASLD, impaired autophagy exacerbates the lipid accumulation and exacerbates oxidative stress (78, 79). EA has been shown to restore hepatic autophagy activity through activation of the AMPK/mTOR pathway (80, 81). EA-induced phosphorylation of AMPK and inhibition of mTOR facilitated the upregulation of autophagy-related proteins such as LC3-II and Beclin-1, as well as enhanced autophagosome formation observed by transmission electron microscopy (73). Furthermore, EA promoted fatty acid oxidation through upregulation of PPAR-α and carnitine palmitoyltransferase 1A (CPT1A), while downregulating sterol regulatory element-binding protein 1c (SREBP1c), a key regulator of adipogenesis (73). These molecular changes facilitated lipid clearance and reduced hepatocellular stress, contributing to the alleviation of liver fibrosis (73). Interestingly, the hepatoprotective effects of EA were significantly diminished by co-administration of Compound C, an AMPK inhibitor, underscoring the pivotal role of AMPK signaling in mediating EA’s effects on autophagy and fibrosis (73). The anti-inflammatory, anti-fibrotic, and pro-autophagic effects of EA are mechanistically interconnected through shared signaling nodes. The AMPK/mTOR pathway, traditionally recognized for its role in energy sensing and autophagy regulation, also modulates inflammatory responses via cross-talk with NF-κB signaling. AMPK activation suppresses NF-κB transcriptional activity through multiple mechanisms, including SIRT1-dependent deacetylation of the p65 subunit and inhibition of IKK phosphorylation. Similarly, SIRT1 not only deacetylates NF-κB but also regulates autophagy through deacetylation of autophagy-related proteins such as ATG5, ATG7, and LC3. This molecular interplay creates a coordinated response wherein EA simultaneously reduces inflammation, enhances autophagic flux, and attenuates fibrogenesis. The convergence of these pathways at the level of AMPK and SIRT1 underscores their role as master regulators of EA’s pleiotropic effects in MASLD (43, 67).

Collectively, these data suggest that EA exerts a therapeutic role in MASLD not only by correcting metabolic derangements, but also by orchestrating a complex interplay between inflammatory suppression, fibrosis attenuation, and autophagy recovery.

## Clinical studies on electroacupuncture in the treatment of MASLD

Several clinical trials have investigated the efficacy of electroacupuncture (EA) in patients with NAFLD/MASLD, providing preliminary evidence supporting its therapeutic potential. A systematic review and meta-analysis published in 2021, encompassing eight randomized controlled trials with a total of 939 patients, demonstrated that acupuncture (including EA) or acupuncture combined with conventional medicine was superior to conventional medicine alone in improving overall clinical efficacy, liver enzymes (ALT, AST), and lipid profiles (TC, TG, LDL-C) in MASLD patients (82). The meta-analysis also identified ST36 (Zusanli), LR3 (Taichong), ST40 (Fenglong), and SP6 (Sanyinjiao) as the most frequently used acupoints (82). However, the authors emphasized that the included studies had insufficient methodological quality and relatively small sample sizes, highlighting the need for larger, rigorously designed trials to confirm these findings (82). Subsequent studies have addressed some of these limitations, as detailed below.

A multicenter, randomized, sham-controlled trial represents one of the most rigorous studies to evaluate the efficacy of EA in patients with MAFLD. This trial adopted a patient-blinded design, recruiting 144 participants who were randomly assigned to receive EA or sham acupuncture treatment over a period of 12 weeks, followed by a 4-weeks post-treatment observation phase (83). Hepatic fat content was assessed using magnetic resonance imaging–derived proton density fat fraction (MRI-PDFF). Preliminary findings indicated that EA led to a clinically meaningful decrease in hepatic fat accumulation compared to sham acupuncture, supporting the therapeutic value of EA in reducing steatosis (83). Additional improvements were observed in liver stiffness (measured by magnetic resonance elastography), metabolic biomarkers, and patient-reported outcomes, collectively indicating that EA enhances liver function and systemic metabolic homeostasis without causing serious adverse effects (83). Regarding safety, the trial protocol prespecified systematic collection of adverse events, including acupuncture-related events such as needling pain, hematoma, bleeding, and fainting, as well as liver-related events (1). Although the final safety analysis is pending trial completion, the protocol’s rigorous adverse event monitoring represents a significant improvement over previous studies and will provide quantitative safety data essential for clinical translation (1).

Another smaller-scale clinical studies have provided additional insights into the metabolic effects of EA. In a randomized controlled trial involving 60 female patients with MASLD, EA administered at classical acupoints (LR14, LR3, ST36, GB34) over a 6-weeks period significantly reduced serum levels of total cholesterol, triglycerides, and low-density lipoprotein cholesterol (84). Although there was no statistically significant increase in high-density lipoprotein cholesterol, the overall lipid-lowering effect of EA was obvious, suggesting that it has a regulatory effect on systemic lipid metabolism (84). These findings are similar to the mechanism observations in preclinical studies, which EA has been proven to regulate adipogenesis and β-oxidation pathways through the AMPK and SREBP1c signaling pathways.

Moreover, EA appears to confer hepatic benefits comparable to aerobic interval training (AIT), a widely accepted non-pharmacologic intervention for MASLD (85, 86). In a study involving 50 MASLD patients, EA and AIT were compared over a 6-weeks period. Both interventions significantly reduced serum ALT, AST, triglycerides, and C-reactive protein (CRP) (77, 87). Notably, EA produced a more pronounced reduction in liver enzymes and triglycerides, suggesting that its effects may be mediated by both anti-inflammatory and metabolic pathways (87).

Despite these promising findings, significant heterogeneity across clinical studies limits the generalizability and comparability of results. Key sources of heterogeneity include variations in acupoint selection–with some studies using fixed classical acupoints (e.g., ST36, LR3, CV12) while others employ individualized point selection based on Traditional Chinese Medicine pattern differentiation (82)–and in stimulation parameters, where electroacupuncture frequency ranges from 2 to 100 Hz with variable pulse widths, intensities, and treatment durations (ranging from 20 to 45 min per session) (23, 44). Additionally, treatment regimens differ, as the number of sessions varies from 18 to 36 over 6–12 weeks, and some studies combine EA with lifestyle modification while others do not (23, 82). Control interventions also vary, including sham acupuncture, no treatment, conventional medication, or lifestyle modification alone, complicating direct comparisons across trials (82). Furthermore, outcome measures are inconsistent; while recent studies have adopted MRI-PDFF as a robust primary endpoint, earlier studies relied on ultrasound or serum biomarkers with variable diagnostic criteria (82, 83). This heterogeneity underscores the need for standardized treatment protocols and core outcome sets to facilitate evidence synthesis and guideline development.

The clinical efficacy of EA has also been extended to patients with NASH, the progressive inflammatory subtype of MASLD characterized by a high risk of fibrosis. In a double-blinded randomized trial involving 60 NASH patients, EA led to a median 33.6% reduction in liver fat content after 12 weeks, significantly outperforming sham acupuncture (88). However, as acknowledged by the authors, this study was designed as a pilot trial with a relatively small sample size and short intervention duration, limiting the generalizability of findings and precluding definitive conclusions regarding long-term efficacy. The study did not assess histological endpoints, which remain the gold standard for NASH trials, and liver stiffness measurements did not show significant changes within the 12-weeks intervention window (88). These limitations highlight the need for larger Phase III trials with longer follow-up periods and histological confirmation to validate EA’s therapeutic efficacy in NASH. Over half of the participants in the EA group achieved a ≥30% reduction in liver fat, a threshold associated with histological improvement in steatohepatitis (88–90). In addition, EA significantly reduced body weight and body mass index (BMI) (88). Although liver stiffness measurements did not significantly change within the short intervention window, the downward trend in serum AST and modest improvements in glucose and lipid parameters emphasized the therapeutic effect of EA on NASH (88).

Importantly, across all clinical trials reviewed, EA exhibited a favorable safety profile with no reports of serious adverse events. This reinforces its feasibility as a non-pharmacological intervention suitable for long-term management of chronic liver conditions. Nonetheless, the heterogeneity in acupuncture protocols, patient populations, and outcome measures remains challenges for standardization and wide clinical application. Future large-scale, multicenter trials are essential to validate current findings and facilitate the integration of EA into clinical practice.

## Safety profile across clinical studies

Across all reviewed clinical trials, EA demonstrated a favorable safety profile. In the largest multicenter trial to date (*n* = 144), the study protocol systematically recorded adverse events including needling pain, hematoma, bleeding, fainting, and local infection, with serious adverse events defined as those requiring hospitalization or resulting in persistent disability (1). Preliminary safety data from the pilot NASH trial (*n* = 60) reported no serious adverse events related to EA; the most common minor adverse events were transient needling pain (reported in 8.3% of participants) and minor subcutaneous hematoma (5.0%), all of which resolved spontaneously without intervention (88). Similarly, Taha et al. reported no significant adverse events in their 60-patient trial (84), and Draz et al. noted that EA was well-tolerated with no dropouts due to adverse effects (87). The meta-analysis by Chen et al. concluded that the safety profile of acupuncture therapy was satisfactory across included studies, although they noted that adverse event reporting was inconsistent and often incomplete (82). These findings reinforce EA’s feasibility as a non-pharmacological intervention suitable for long-term management of chronic liver conditions. However, standardized adverse event reporting using validated instruments (e.g., the Acupuncture Adverse Events Checklist) should be implemented in future trials to enable quantitative safety meta-analysis.

## Challenges, limitations, and future directions of EA in MASLD management

Despite the promising therapeutic potential of electroacupuncture (EA) in the treatment of NAFLD/MASLD, several significant challenges and limitations currently hinder its widespread clinical adoption and acceptance within mainstream hepatology practice. A primary obstacle to evidence synthesis and clinical translation is the substantial heterogeneity across existing studies, which manifests across multiple domains including acupoint selection, stimulation parameters, treatment regimens, control interventions, and outcome measures (23, 32, 44, 82). Protocols vary considerably, with some studies using fixed classical acupoints such as ST36, LR3, and CV12 while others employ individualized point selection based on Traditional Chinese Medicine pattern differentiation (82). Electroacupuncture frequency ranges from 2 to 100 Hz, with variable pulse widths, intensities, and treatment durations spanning 20–45 min per session (32, 44, 82). The number of sessions varies from 18 to 36 over 6 to 12 weeks, and some studies combine EA with lifestyle modification while others do not (23, 82). Control interventions include sham acupuncture, no treatment, conventional medication, or lifestyle modification alone, further complicating direct comparisons across trials (82). While recent studies have adopted MRI-PDFF as a robust primary endpoint, earlier studies relied on ultrasound or serum biomarkers with variable diagnostic criteria (82, 83, 88). This heterogeneity precludes meaningful meta-analysis and impedes the development of evidence-based clinical guidelines. Future trials must adhere to standardized reporting guidelines such as the STRICTA checklist, which provides comprehensive specifications for reporting acupuncture interventions, including rationale, needling details, treatment regimen, practitioner background, and control interventions (91).

The interpretation of acupuncture clinical trials is further complicated by the ongoing debate regarding the nature and magnitude of placebo effects. Sham acupuncture controls–which may involve needling at non-acupoints, superficial needling, or non-penetrating sham devices–consistently produce clinically meaningful improvements in various conditions, raising questions about the specific efficacy of verum acupuncture (92). A recent systematic review and meta-analysis of sham acupuncture in insomnia patients demonstrated that sham acupuncture produced significant improvements from baseline, with low-intensity stimulation and medium-frequency treatment associated with stronger placebo effects (93). The network meta-analysis revealed that sham acupuncture performed at non-meridian, non-acupoint locations using low-intensity stimulation produced the greatest placebo effects (93). These findings have direct implications for MASLD trials. While the multicenter trial by Zhao et al. incorporates a sham acupuncture control with needles inserted at non-acupoints using a non-penetrating device (83), the potential for substantial placebo responses necessitates careful interpretation of between-group differences. Mechanistically, acupuncture activates the pain matrix via spinothalamic pathways, and these central nervous system effects can be modulated through patient expectations–a central component of placebo responses (92). Future MASLD trials should incorporate rigorous blinding assessment, measure expectancy and credibility, and consider employing multiple control conditions to disentangle specific from non-specific effects (92, 93).

Beyond these methodological considerations, the existing clinical evidence for EA in MASLD suffers from several inherent limitations that must be acknowledged. Sample sizes remain small, with the largest published trial to date including only 60–144 participants, limiting statistical power and generalizability (83, 88). Intervention duration is typically short, with most studies employing 6–12 weeks treatment periods and limited follow-up, precluding assessment of long-term durability and fibrosis regression (84, 87, 88). Critically, no published EA trial has utilized liver biopsy–the gold standard for NASH diagnosis and fibrosis staging–as a primary outcome measure (88). While MRI-PDFF has emerged as a validated non-invasive biomarker for steatosis, its correlation with histological improvement in inflammation and ballooning is imperfect (89, 90). Safety reporting remains inconsistent across studies; although EA appears generally safe, quantitative adverse event data with standardized reporting instruments are lacking (82). The ongoing multicenter trial by Zhao et al. addresses several of these limitations through systematic adverse event monitoring, including needling pain, hematoma, bleeding, fainting, and local infection (83). Preliminary safety data from existing trials report no serious adverse events related to EA, with the most common minor adverse events being transient needling pain (reported in 8.3% of participants) and minor subcutaneous hematoma (5.0%), all of which resolved spontaneously without intervention (88). Similar findings have been reported in other trials, with EA consistently demonstrating a favorable safety profile (84, 87). However, definitive Phase III trials with histological endpoints and long-term follow-up remain urgently needed to establish the efficacy and safety of EA for MASLD management.

To properly contextualize EA’s therapeutic potential, direct comparison with established interventions is essential. Lifestyle modification–including dietary interventions, weight loss, and increased physical activity–remains the cornerstone of MASLD management, with sustained 7%–10% weight loss demonstrating improvement in steatosis, inflammation, and even fibrosis (19). However, long-term adherence to lifestyle modifications is often limited, with high rates of weight regain and poor sustainability (19). Pharmacological options have recently expanded with the FDA approval of resmetirom–a thyroid hormone receptor-β agonist–for non-cirrhotic MASH with moderate to advanced fibrosis (6). Additionally, GLP-1 receptor agonists such as semaglutide have shown promise in improving steatohepatitis (20). Compared to these interventions, EA offers several potential advantages. The therapy demonstrates a favorable safety profile, as unlike pharmacotherapies with potential hepatotoxicity or systemic side effects, EA-related adverse events are typically mild and transient (83, 84, 87). EA simultaneously targets multiple pathogenic pathways–including lipid metabolism, insulin sensitivity, inflammation, and gut barrier function–through integrated neuro-endocrine-immune mechanisms, offering a multisystem approach that single-target pharmacotherapies cannot provide (43, 44). Furthermore, acupuncture is well-accepted in many cultural contexts and may appeal to patients seeking non-pharmacological approaches (94). However, direct comparative effectiveness trials evaluating EA against or in combination with lifestyle modification and pharmacotherapy are lacking and represent a critical research priority.

Beyond questions of efficacy, several practical barriers impede the integration of EA into routine clinical practice for MASLD management. The practice of medical acupuncture requires specialized training, and regulatory frameworks vary substantially across jurisdictions, affecting practitioner availability and quality assurance (94, 95). Treatment accessibility and cost present additional challenges, as EA typically requires multiple sessions per week over several weeks, imposing significant time and financial burdens on patients. Reimbursement policies for acupuncture vary widely across healthcare systems, limiting equitable access (94). While serious infections from acupuncture are rare when proper aseptic techniques are employed, concerns persist regarding needling-related infections, particularly in patients with chronic liver disease who may have altered immune function or increased bleeding risk (42). Effective implementation also requires interprofessional collaboration between acupuncturists, hepatologists, primary care physicians, and dietitians, yet such integrated care models remain underdeveloped (94, 95). Addressing these implementation barriers will require development of standardized training curricula and credentialing pathways, health economic evaluations assessing cost-effectiveness, establishment of clear infection control protocols, and creation of integrated care pathways that position EA as a complementary rather than alternative therapy (94–96).

Based on the identified gaps and challenges, future research should prioritize several strategic directions. Large-scale, multicenter Phase III trials with rigorous design, adequate sample sizes, long-term follow-up extending to at least 12 months, and histological endpoints are urgently needed to establish definitive efficacy (83, 88). Such trials should employ standardized treatment protocols based on systematic reviews of optimal acupoint selection and stimulation parameters (82). While preclinical mechanisms are increasingly well-characterized, translational studies incorporating human liver tissue, advanced imaging biomarkers, and systems biology approaches are needed to validate proposed pathways–including AMPK/SIRT1 and Pdia3/Perk/Qrich1 signaling–in patients (42–44). Investigating synergistic effects of EA combined with lifestyle modification, resmetirom, GLP-1 agonists, or metformin may identify optimal integrative treatment strategies (20, 73). Identifying patient characteristics–such as genetic polymorphisms, metabolic phenotypes, and gut microbiome profiles–that predict differential responses to EA could enable precision prescription and personalized medicine approaches (23, 44). Health services and implementation research evaluating cost-effectiveness, patient preferences, barriers to access, and optimal integration models within existing healthcare systems is essential for successful translation (94, 95). Finally, implementation of validated safety monitoring instruments across all future trials will facilitate quantitative safety meta-analysis and inform clinical risk-benefit assessment (82, 88).

In summary, while EA represents a promising adjunctive therapy for MASLD with multifaceted mechanistic effects and a favorable safety profile, substantial methodological, evidence-based, and implementation challenges must be addressed before it can be widely recommended in clinical practice. Addressing these challenges through rigorous research and systematic implementation efforts will determine whether EA fulfills its potential as a valuable component of comprehensive MASLD management.

## Conclusion

Electroacupuncture (EA) represents a novel and promising adjunctive therapy for metabolic dysfunction-associated steatotic liver disease (MASLD), exerting beneficial effects through multifaceted modulation of metabolic, inflammatory, and fibrotic pathways (1, 44, 73). By targeting key signaling cascades such as AMPK/mTOR, SIRT1/NF-κB, and PI3K/Akt, EA improves hepatic lipid metabolism, insulin sensitivity, and liver function (42, 43, 73). These pathways do not operate in isolation but rather constitute an integrated “neuro-endocrine-immune-metabolic” regulatory network, wherein EA at specific acupoints triggers coordinated central and peripheral responses that collectively ameliorate hepatic steatosis and systemic metabolic dysfunction (43, 44). Emerging evidence further expands this mechanistic understanding, demonstrating that EA activates the NRF2/ARE pathway to attenuate oxidative stress and cuproptosis–a newly recognized form of programmed cell death implicated in MASLD pathogenesis. Additionally, EA modulates the gut-liver axis through α7nAChR-mediated preservation of intestinal barrier integrity, reducing endotoxin translocation and hepatic inflammation (44). Table 1 shows the summary of the studies included in the review.

**TABLE 1:** Summary of the studies included in the review.

MASLD model	Species	Intervention methods	Points used	Results	References
MAFLD	Patients	EA	CV12, CV4, ST25, SP15, LR13, LI4, ST36, SP6 and LR3.	EA reduced liver fat content in patients with MAFLD.	(83)
MASLD	Patients	EA	LR14, LR3, ST36, GB34	EA decreased the expression of LDL, TC, TG in MASLD patients.	(84)
MASLD	Patients	EA	LR14, LR3, ST36, GB34	EA can improve the liver function of patients with MASLD better than AIT exercise.	(28)
NASH	Patients	EA	CV12, CV4, bilateral ST25, SP15, LV13, ST36, SP6, LI4, and LV3.	EA effectively and safely reduced liver fat content in NASH patients.	(88)
MASLD	Rats	EA	FengLongXue, YinLingQuanXue, SanYinJiaoXue.	EA significantly alleviated liver inflammatory reaction of MASLD by enhancing SIRT1 expression and suppressing NLRP3/NF-kB signal pathway.	(67)
MAFLD	Rats	EA	GB26, ST36, ST40, CV12.	EA improved reduced oxidative stress and ectopic fat deposition of ZDF rats.	(28)
MASLD	Rats	EA	Fenglong point	EA improved hyperlipidemia and reduced hepatic cholesterol synthesis by inhibiting the phosphorylation of USP20 and mTOR and the expression of HMGCR.	(35)
MASLD	Rats	EA	BL 19, EX B3, ST 36, SP 6.	EA exerted its effect in improving liver steatosis and fibrosis by improving glycolipid metabolism and autophagy through the promotion of the PPAR-α/CPT1A and AMPK/mTOR pathways expression.	(73)
MASLD	Rats	EA	CV3, CV4 and ST40.	EA significantly reduced free fatty acids, TG, TC and LDL by promoting adiponectin, AMPKα1, LKB1 expression and inhibiting ACC expression in obese rat model of PCOS.	(34)
MASLD	Rats	EA	ST25, ST36.	EA significantly reduced fasting blood glucose, fasting insulin, homeostasis model assessment of IR indices, and triglycerides, and alleviated hepatic steatosis by downregulating the expression of TNF-α and IL-6, upregulating the expression of PPAR-α and PI3K/AKT insulin signaling pathway in the liver.	(38)
MASLD	Rats	EA	ST36	EA effectively reversed Olanzapine-induced hepatocyte damage, and restored disturbances in free fatty acids by deactivating AGRP-expressing neurons in the LS, NAc and pallidum.	(41)
MASLD	Mice	EA	BL13, BL20, BL23, LI4, LR3, ST36, SP6	EA significantly reduced the steatoticdegeneration of mice hepatocytes by reducing TG, LDL, CHO, and INS levels and increasing the HDL level through the regulation of AMPK/ACC and PI3K/AKT signaling pathway.	(32)
MASLD	Rats	EA	ST36, ST44.	EA reduced high-fat diet-induced hepatic lipid accumulation by regulating AMPK/ACC signaling pathways.	(33)
MASLD	Rats	EA	ST36, SP6.	EA protected the function of hepatic cells by ameliorating insulin resistance via AMPK/TSC2//mTORC1/p70S6K pathway.	(47)
MASLD	Rats	EA	ST36, PC6, SP6, BL18, ST40.	EA improved hepatic steatosis by inhibiting the expression of Pdia3/Perk/Qrich1 signal pathway.	(42)

The studies summarized above employed heterogeneous outcome measures, limiting direct comparability. Key efficacy endpoints across studies included: (1) Hepatic steatosis: assessed by MRI-PDFF (percentage reduction) in recent trials (83, 88), or by ultrasound (steatosis grade) and liver fat content (quantitative measurement) in earlier studies (28, 84); (2) Liver fibrosis: evaluated by magnetic resonance elastography (liver stiffness measurement in kPa) (83) or histological fibrosis score in animal studies (73); (3) Liver enzymes: serum ALT and AST levels (U/L) (67, 73, 87); (4) Lipid profiles: TC, TG, LDL-C, and HDL-C (mmol/L or mg/dL) (32, 34, 84); (5) Glucose metabolism: Fasting glucose, fasting insulin, and HOMA-IR (38, 47); (6) Inflammatory markers: TNF-α, IL-6, and CRP (38, 67, 87); (7) Anthropometric parameters: body weight, BMI, and waist circumference (84, 87, 88). Future studies should prioritize standardized core outcome sets, with MRI-PDFF as the preferred non-invasive measure of hepatic steatosis and liver stiffness measurement by elastography for fibrosis assessment, to enhance cross-trial comparability and facilitate meta-analysis (83, 88).

When contextualized within the current therapeutic landscape, EA offers distinct advantages. Lifestyle modification remains the cornerstone of MASLD management but suffers from poor long-term adherence (19), while pharmacological options–including the recently approved resmetirom and GLP-1 receptor agonists–carry potential systemic side effects (6, 20). In contrast, EA demonstrates a favorable safety profile with typically mild and transient adverse events (84, 87, 88) and simultaneously targets multiple pathogenic pathways through integrated neuro-endocrine-immune mechanisms (43, 44). Notably, preclinical evidence suggests synergistic effects when EA is combined with metformin, significantly improving hepatic AMPK expression, liver morphology, and fibrosis compared to either intervention alone (73), positioning EA as a potential adjunct to pharmacotherapy in comprehensive MASLD management.

Despite these encouraging findings, several challenges must be addressed before EA can be widely recommended in clinical practice. Substantial heterogeneity across existing studies–in acupoint selection, stimulation parameters, treatment regimens, and outcome measures–limits evidence synthesis and guideline development (82). The influence of placebo effects in acupuncture trials remains a contentious issue requiring rigorous blinding assessment. Furthermore, practical barriers including acupuncture training requirements, treatment accessibility, cost considerations, and the need for interprofessional collaboration must be systematically addressed through implementation science approaches (94, 95).

Future research should prioritize large-scale, multicenter Phase III trials with histological endpoints, long-term follow-up, and standardized treatment protocols (82, 83, 88). Translational studies incorporating human liver tissue are needed to validate preclinical mechanistic findings, particularly for pathways such as Pdia3/Perk/Qrich1 signaling currently demonstrated only in animal models (42). Investigating personalized medicine approaches based on patient characteristics–including genetic polymorphisms, metabolic phenotypes, and gut microbiome profiles–may enable precision prescription and optimize therapeutic outcomes (43, 44). Health economic evaluations and implementation research assessing cost-effectiveness and optimal integration models within existing healthcare systems are essential for successful clinical translation (94, 95).

In summary, EA represents a promising adjunctive therapy for MASLD with multifaceted mechanistic effects targeting lipid metabolism, insulin sensitivity, inflammation, oxidative stress, autophagy, and gut barrier function through integrated neuro-endocrine-immune pathways. Its favorable safety profile, potential for synergistic combination with pharmacotherapy, and patient acceptability in diverse cultural contexts support continued investigation. Addressing current methodological, evidence-based, and implementation challenges through rigorous research will determine whether EA fulfills its potential as a valuable component of comprehensive, personalized MASLD management.
